# Supplementary material for: Characterization and Sequence Mapping of Large RNA and mRNA Therapeutics Using Mass Spectrometry
Source: Anal Chem. 2022 May 12;94(20):7339–49. doi: 10.1021/acs.analchem.2c00765 (PMC9134182; doi:10.1021/acs.analchem.2c00765)
Supplement: Supplementary file 1 — ac2c00765_si_001.pdf [file ac2c00765_si_001.pdf]

Supporting Information

Characterisation and sequence mapping of large RNA and mRNA therapeutics using mass spectrometry

Christina J. Vanhinsbergh[1], Angela Criscuolo[2], Jennifer N. Sutton[4], Keeley Murphy[4], Andrew J.K. Williamson[3], Ken Cook[3] and Mark J. Dickman[1]*

[1] Department of Chemical & Biological Engineering, University of Sheffield, Sheffield, S1 3JD, UK

[2] ThermoFisher Scientific GmbH, Dreieich, Germany, DE 63303

[3]ThermoFisher Scientific, Hemel Hempstead, Hertfordshire, HP2 7GE, UK

[4] ThermoFisher Scientific, San Jose, California, USA

Table of Content:

Figure S1 IP RP HPLC analysis of intact RNA S2

Figure S2 Base Peak Chromatograms of the partial RNase T1 digests of RNA S3

Figure S3 Identification of sequence isomers from the partial RNase T1 digest of the SARS CoV-2 spike protein mRNA S4

Figure S4 MS and MS/MS spectra of selected oligoribonucleotides from the partial RNase T1 digest of SARS CoV-2 spike protein mRNA S5

Figure S5 Total Ion Chromatograms of the complete RNase T1 digests of RNA S6

Figure S6 Bar chart of the % sequence coverage of the SARS CoV-2 spike protein mRNA S7

Table S1. Identified oligoribonucleotides from the partial RNase T1 digests S8

Table S2. Identified oligoribonucleotides from the complete in solution RNase T1 digests S8

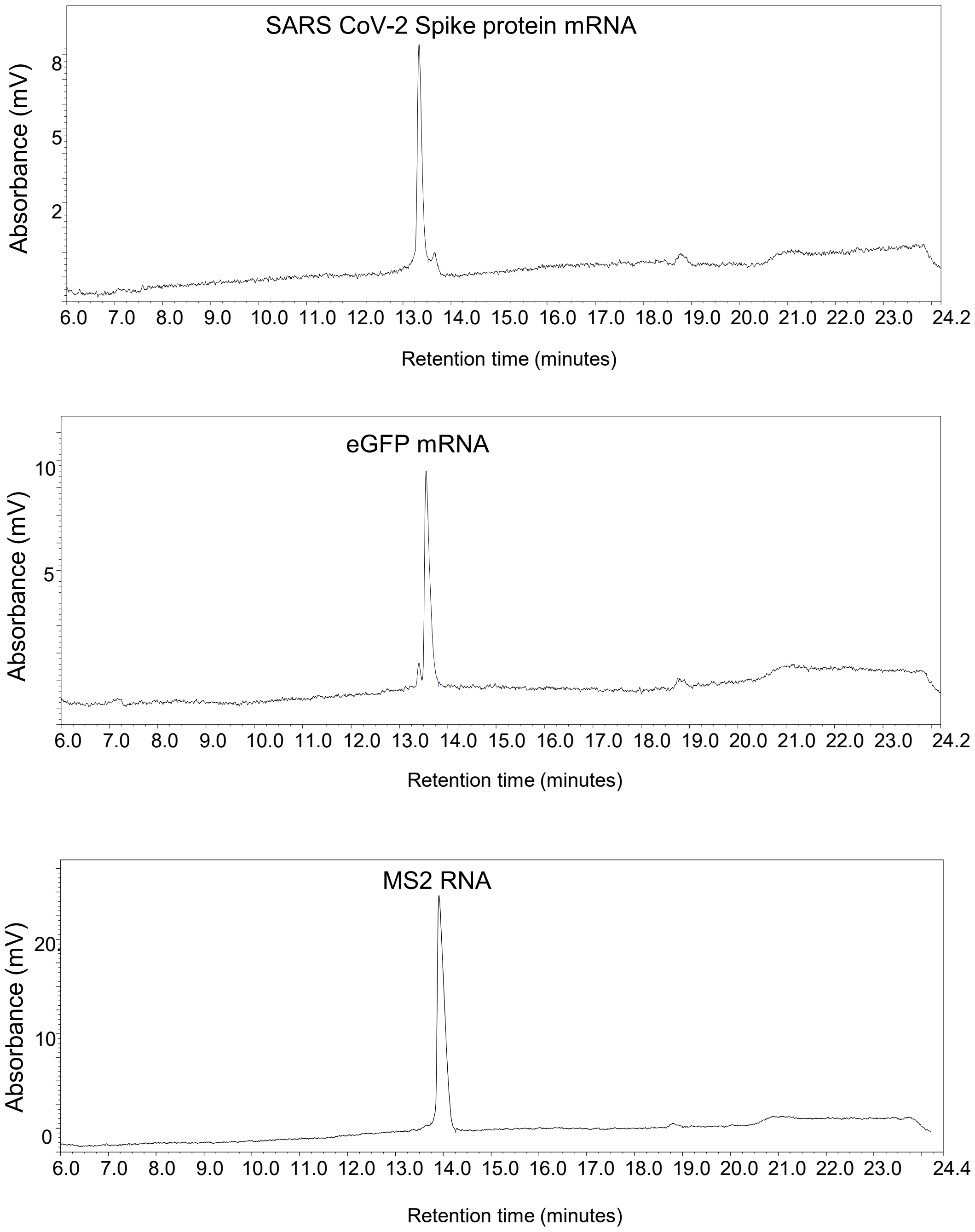

Figure S1. IP RP HPLC analysis of intact RNA. A) Chromatogram shows the analysis of the SARS CoV-2 Spike protein mRNA following synthesis using IVT and purification. B) Chromatogram show the analysis of the eGFP mRNA following synthesis using IVT and purification. C) Chromatogram of the MS2 RNA. 100 ng of each RNA was analysed using IP RP HPLC with UV detection at 260 nm.

Figure S1

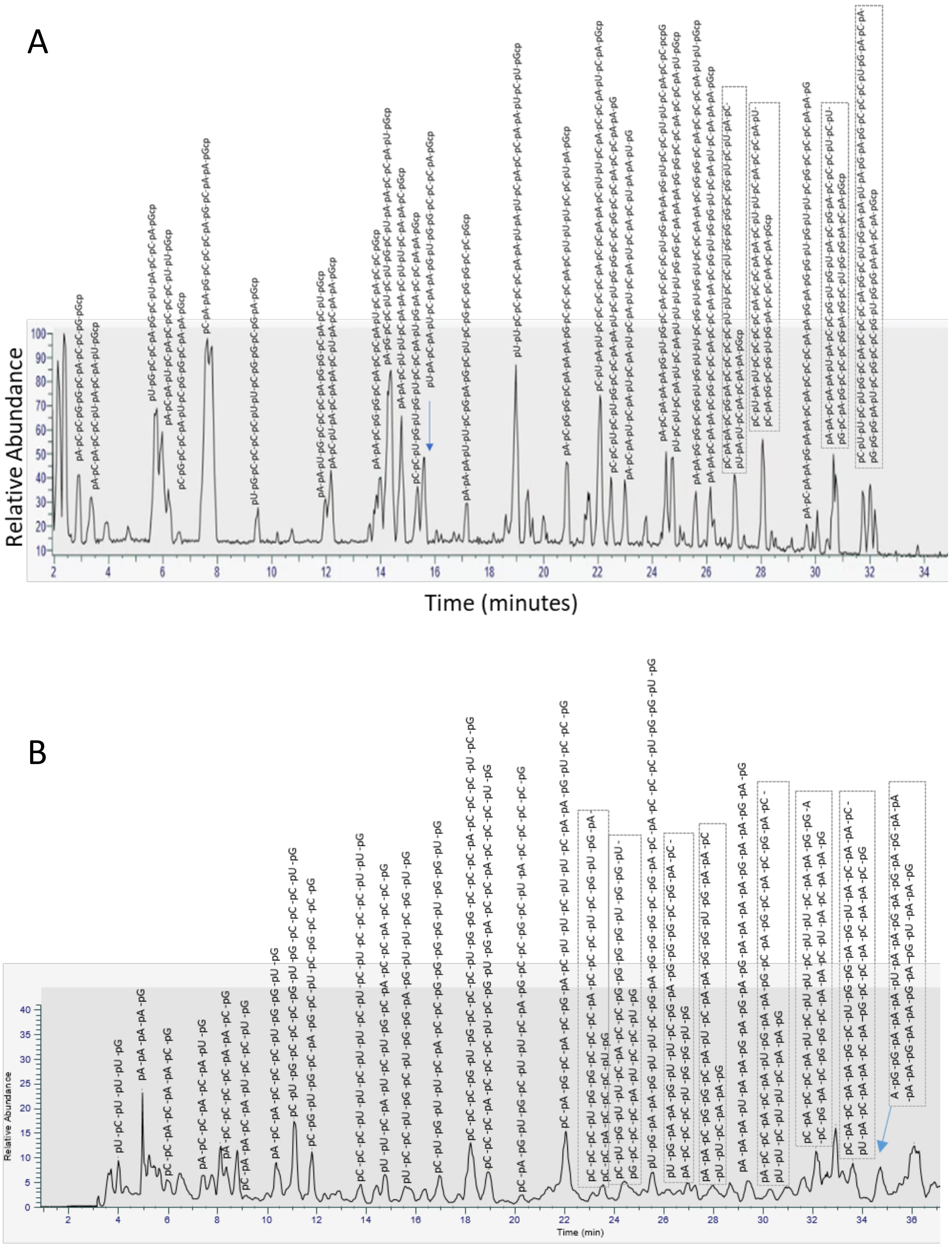

Figure S2. Base Peak Chromatograms of the partial RNase T1 digests of RNA. IP RP HPLC in conjunction with MS analysis was used to analyse partial RNase T1 digests of A) SARS CoV-2 spike protein mRNA. B) eGFP mRNA. Selected identified oligoribonucleotides are highlighted.

Figure S2

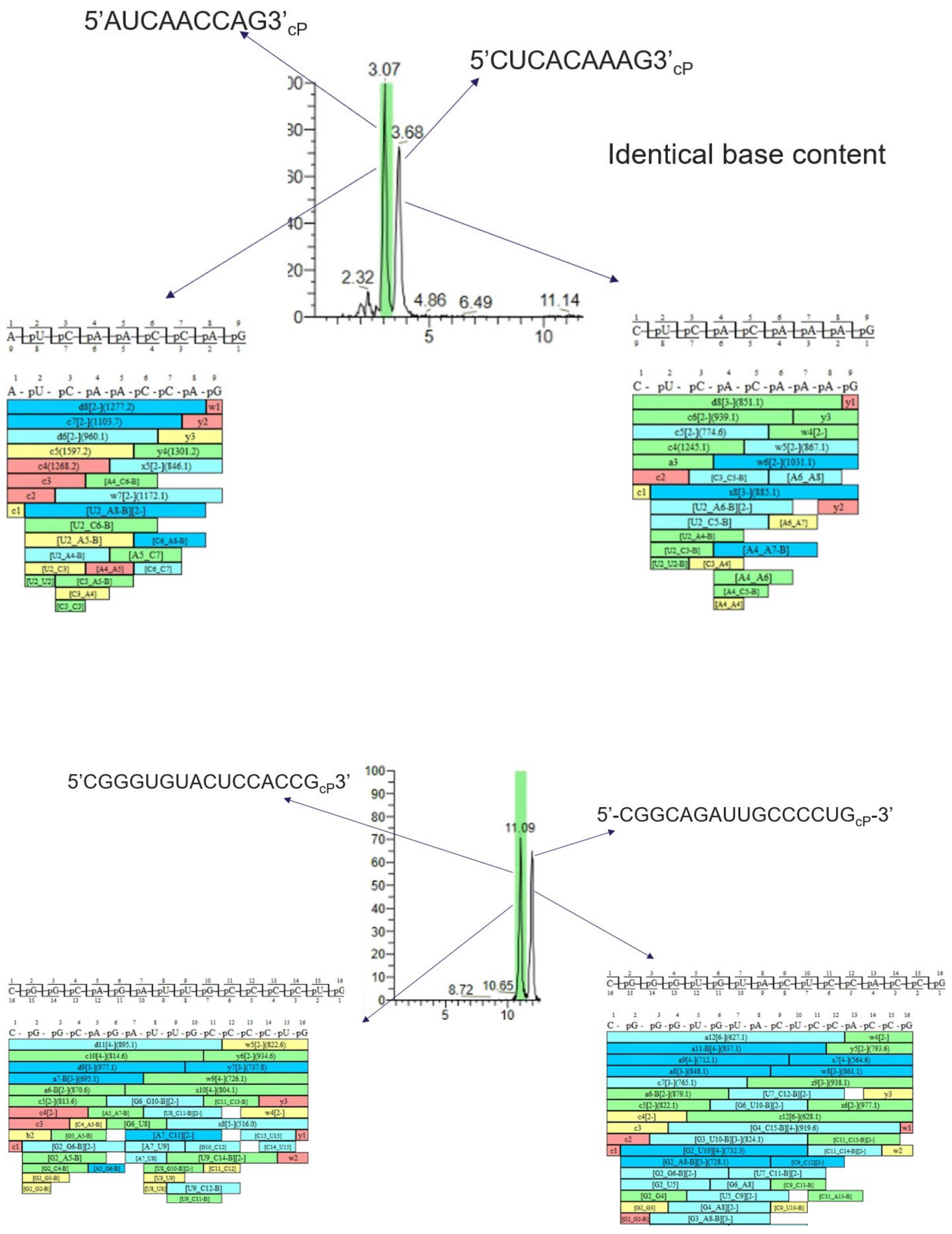

Figure S3. Identification of sequence isomers from the partial RNase T1 digest of the SARS CoV-2 spike protein mRNA. Oligonucleotide sequence isomers containing the same base composition were typically separated during the chromatography and the MS² fragmentation data with the automated sequence annotation enabled identification of sequence isomers.

Figure S3

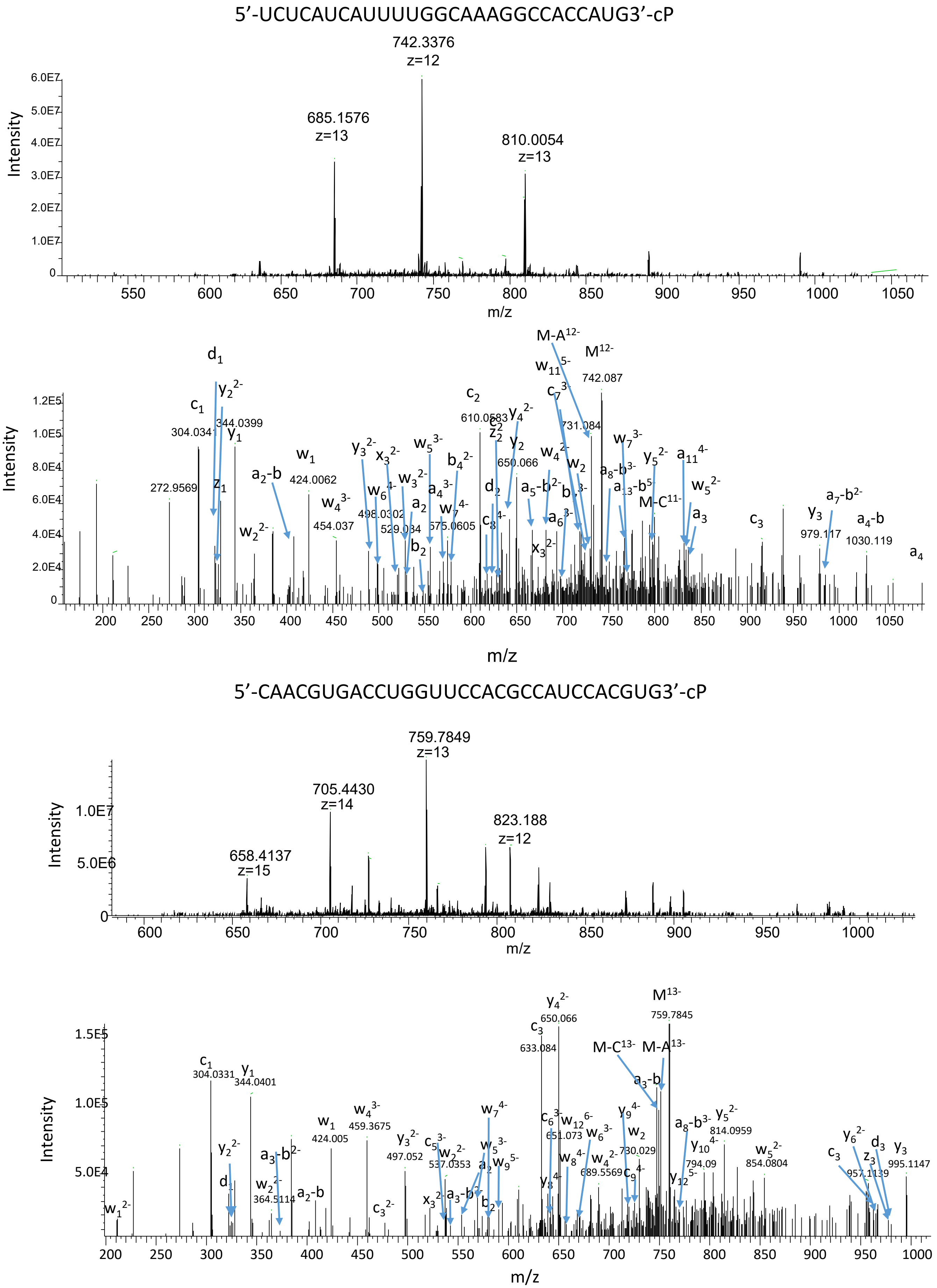

Figure S4. MS and MS/MS spectra of selected oligoribonucleotides from the partial RNase T1 digest of SARS CoV-2 spike protein mRNA. The corresponding fragment ions are highlighted. Internal fragments have not been highlighted for clarity

Figure S4

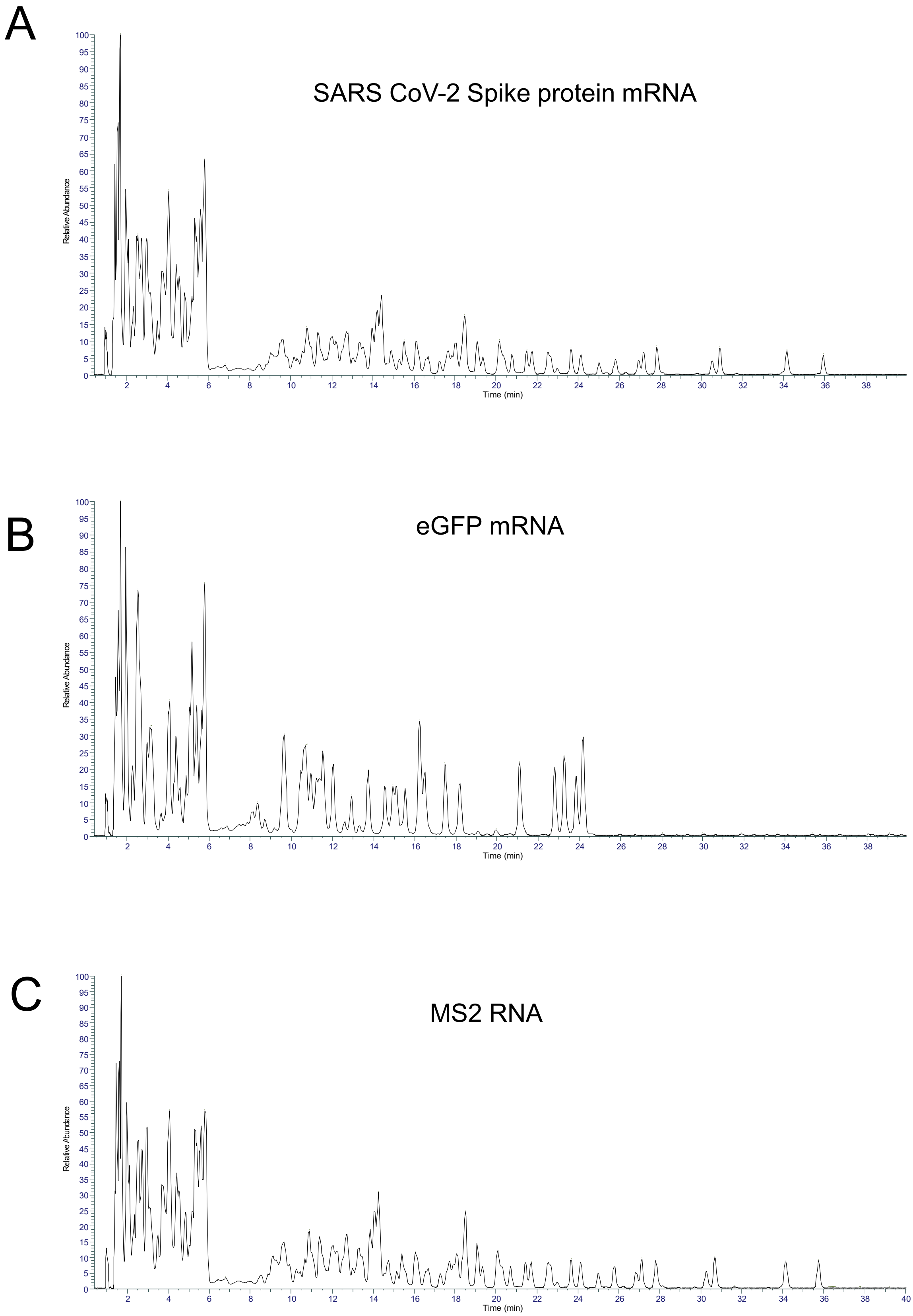

Figure S5. Total Ion Chromatograms of the complete RNase T1 digests of RNA. IP RP HPLC in conjunction with MS analysis was used to analyse complete T1 digests of A) SARS CoV-2 spike protein mRNA B) eGFP mRNA and C) MS2 RNA. In each RNase T1 digest 10 µg of RNA was incubated with RNase T1 for 4 hours at 37 °C.

Figure S5

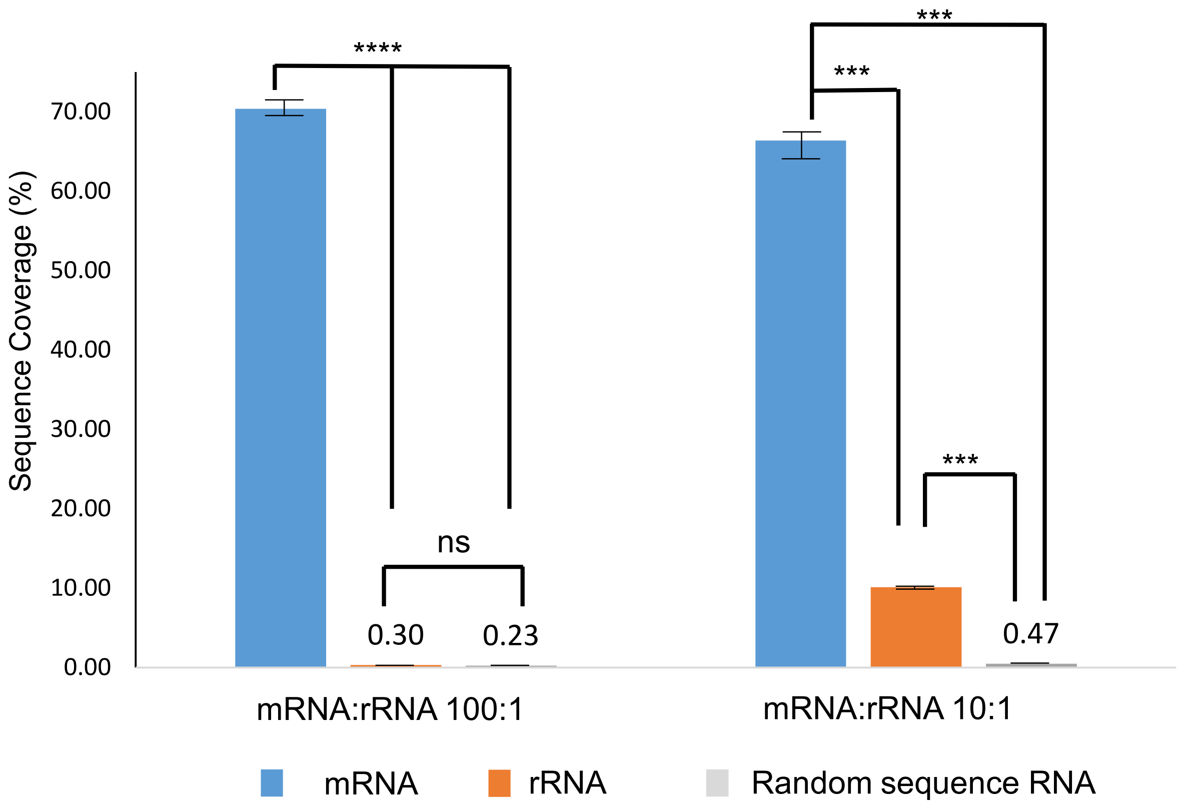

Figure S6. Bar chart of the % sequence coverage of the SARS CoV-2 spike protein mRNA, rRNA and random sequence RNA. The mRNA and rRNA were mixed in either 100:1 or 10:1 mass ratio prior to partial RNase T1 digestion and LC MS/MS analysis. In all graphs, mean and S.E. are plotted (n=3). Stars denote significance as calculated by T-tests. ns = P > 0.05, * = P ≤ 0.05, ** = P ≤ 0.01, *** = P ≤ 0.001, **** = P ≤ 0.0001

Figure S6

Table S1. Identified oligoribonucleotides from the partial RNase T1 digests

Table S2. Identified oligoribonucleotides from the complete in solution RNase T1 digests
